# Perioperative Anaesthetic Management for Laparoscopic Gastrectomy in a Patient with Coarctation of Aorta

**DOI:** 10.7759/cureus.7224

**Published:** 2020-03-09

**Authors:** Maha Jahangir, Abdullah Imtiaz, Athar Siddiqui, Shahid Khattak, Danish Imtiaz

**Affiliations:** 1 Anesthesiology, Dow Medical College, Karachi, PAK; 2 Internal Medicine, Sheikh Khalifa Bin Zayed Al-Nahyan Hospital, Lahore, PAK; 3 Anesthesiology, Shaukat Khanum Memorial Cancer Hospital and Research Centre, Lahore, PAK; 4 Surgical Oncology, Shaukat Khanum Memorial Cancer Hospital and Research Centre, Lahore, PAK

**Keywords:** coarctation of aorta, anaesthetic management, laparascopic gastrectomy, oesophageal anastomosis, gastric adenocarcinoma

## Abstract

Coarctation of the aorta (CoA) is a congenital condition, usually diagnosed and corrected early in life. Long-term survival with untreated coarctation is uncommon and is associated with high mortality rates in the fifth decade. A patient with CoA may present with problems while undergoing cardiac or non-cardiac surgical procedures which could pose considerable challenges in their anaesthetic management. Hence, the choice of anaesthetic technique plays an important role in determining the perioperative course and postoperative outcome in patients with CoA. This report discusses a case of middle-age man, recently diagnosed with CoA while undergoing a preanaesthetic assessment prior to the surgery for gastric adenocarcinoma involving proximal gastro-oesophageal junction. It highlights the successful anaesthetic management of CoA scheduled for laparoscopic-assisted gastrectomy for gastric adenocarcinoma. Perioperative management goals of the patient included general anaesthesia, epidural analgesia to avoid pain-associated adverse effects and efficient control of blood pressure distal to coarctation to limit the risk of intraoperative morbidity. It also demonstrates a major impact on anaesthesiologists who serve the most important role in managing such patients undergoing surgery with ‘red flag’ features.

## Introduction

Coarctation of the aorta (CoA) is a congenital condition in which the aorta is narrow, usually in the area where the ductus arteriosus inserts, just distal to the left subclavian artery. It is found in approximately 4% to 6% of all patients with congenital heart defects, with a 2:1 predominance in males [1,2]. Untreated CoA is uncommon in the fifth decade of life and is associated with 75% and 90% mortality by 46 and 58 years of age, respectively [1,3]. A patient with CoA may present with problems during cardiac or non-cardiac surgical procedures which could pose considerable challenges in their anaesthetic management. The choice of anaesthetic technique plays an important role in determining the perioperative course and postoperative outcome in patients with CoA. Applying a team-based approach among the cardiologist, anaesthesiologist and surgeon is important to determine the perioperative plan to mitigate the cardiovascular risk.

The literature regarding perioperative anaesthetic management of major gastro-oesophageal resection in a patient with CoA is scarce. We present a case of successful perioperative anaesthetic management of CoA scheduled for laparoscopic-assisted gastrectomy for gastric adenocarcinoma. 

## Case presentation

A 57-year-old male was diagnosed as a case of proximal gastric adenocarcinoma, 42 cm from incisors involving the gastro-oesophageal junction by oesophagogastroduodenoscopy and biopsy, staging CT and endoscopic ultrasound. Staging laparoscopy confirmed T3N1 stage of cancer. The patient was discussed in the gastrointestinal multidisciplinary team meeting and subsequently planned for perioperative chemotherapy followed by resection. 

He was recently diagnosed with CoA with mild dilatation of aortic root and mild aortic stenosis while undergoing a preoperative assessment prior to the surgery. The patient denied any history of chest pain, palpitations, syncope, tachycardia or headache. However, there were visible pulsations on the anterior and lateral chest wall which he denied taking notice of. Radial-femoral arterial delay was present. Electrocardiogram showed normal sinus rhythm and left ventricular hypertrophy. Cardiology input stated that the patient did not need any intervention for CoA immediately. A detailed discussion with the patient and anaesthesia team was held after which it was decided to avoid thoracotomy, as the patient had distended intercostal vessels which would increase the perioperative morbidity. After explaining the benefits and risks and taking informed consent from the patient, he was scheduled for elective laparoscopic-assisted proximal gastro-oesophageal resection.

His weight and height were 60 kg and 5.54 feet, respectively (body mass index: 21.0). Cardiac auscultation revealed an ejection systolic murmur of grade 2/6 at the upper sternal border radiating to neck and in the left infraclavicular area. Routine blood and biochemical investigations were within normal limits. Pre-induction vitals were blood pressure (BP) 150/70 mmHg in the right arm, heart rate 65/min with regular sinus rhythm, respiratory rate 20/min and peripheral capillary oxygen saturation (SpO_^2^_) 98% and 94% on room air on the right and left arm, respectively. He belonged to the American Society of Anesthesiologists (ASA) status III. On airway examination, the patient had Mallampati grade II with normal mouth opening and adequate neck extension.

In the operating room, after a formal World Health Organization (WHO) safety checklist, a thoracic epidural catheter was placed midline with the patient in sitting position using an 18G Tuohy needle at T8-T9 interspace as a primary modality for postoperative analgesia. Three millilitres of 2% lidocaine was given as a test dose. The patient was invasively monitored. Preoxygenation was done with 100% O_2_, followed by intravenous (IV) induction of anaesthesia with morphine sulphate 2 mg, propofol 100 mg and intermediate-acting non-depolarizing muscle relaxant atracurium 30 mg. IV boluses of maintenance dose of atracurium 0.1 mg/kg were administered. The patient was ventilated with a bag and mask for three minutes, and a 37 Fr left-sided double-lumen endobronchial tube was placed using a video laryngoscope. Correct position of the bronchial cuff was confirmed using the fiberoptic scope. Two arterial lines were placed and monitored: one in the right radial artery to measure the BP changes before the coarctation, and another in the right dorsalis pedis artery to measure the BP changes after the coarctation and due to carbon dioxide gas insufflation. Central venous catheter was placed in the right internal jugular vein under ultrasound guidance, and two wide bore peripheral IV lines were secured. Ventilation was achieved using volume-controlled ventilation mode with positive end-expiratory pressure of 5 cm H_2_O, tidal volume 7 ml/kg and respiratory rate 12/min during bilateral lung ventilation. Pneumoperitoneum was created, and the pressure was created using carbon dioxide (CO_2_) gas insufflation and maintained at <12 mmHg. 

Intraoperatively, systolic and diastolic BPs were maintained between 100-120 mmHg and 70-85 mmHg, respectively, in both upper and lower limbs, and mean arterial pressure (MAP) was maintained ≥65 mmHg in both arms. Occasional episodes of hypotension were first managed with fluids, and then with 10 mcg boluses of phenylephrine. During five hours of combined anaesthesia and surgery, a total of 3,500 ml of IV crystalloid fluid was infused and urine output was monitored on an hourly basis with total urine output of 200 ml. At the end of the surgery, neuromuscular block was reversed using neostigmine and glycopyrrolate 2.5 mg and 0.5 mg IV injection, respectively. Using nerve stimulator, train of four (ToF) was calculated and reversal agent was given when >0.9. ToF revealed four twitches after reversal. Awake extubation was done, and the patient was shifted to the postanaesthesia care unit (PACU) with supplemental oxygen via a face mask to maintain SpO_2_ >94%, and cardiac monitor attached for invasive monitoring. IV fluid was administered at a rate of 100 ml/hr. Epidural infusion of bupivacaine 0.125% with fentanyl 1 mcg/ml was administered at the rate of 7-10 ml/hr, keeping MAP > 65 mmHg for postoperative analgesia.

PACU stay was uneventful, and the patient was shifted to surgical high dependency unit (HDU). Standard HDU care was given to the patient including deep venous thrombosis and stress ulcer prophylaxis, nutritional support, oral hygiene and wound dressing. The patient was observed overnight and stepped down to the surgical elective facility in a stable condition on the second postoperative day. The patient was kept *nil *per oral, was started on feeding via nasojejunal feeding tube and progressed gradually to full caloric requirement. No postoperative chest complications occurred, and all lines including an epidural catheter, Foley catheter and abdominal drain were gradually removed in the next four days. The patient was discharged in a stable condition on the sixth postoperative day with proper follow-ups advised.

## Discussion

**Figure 1:**
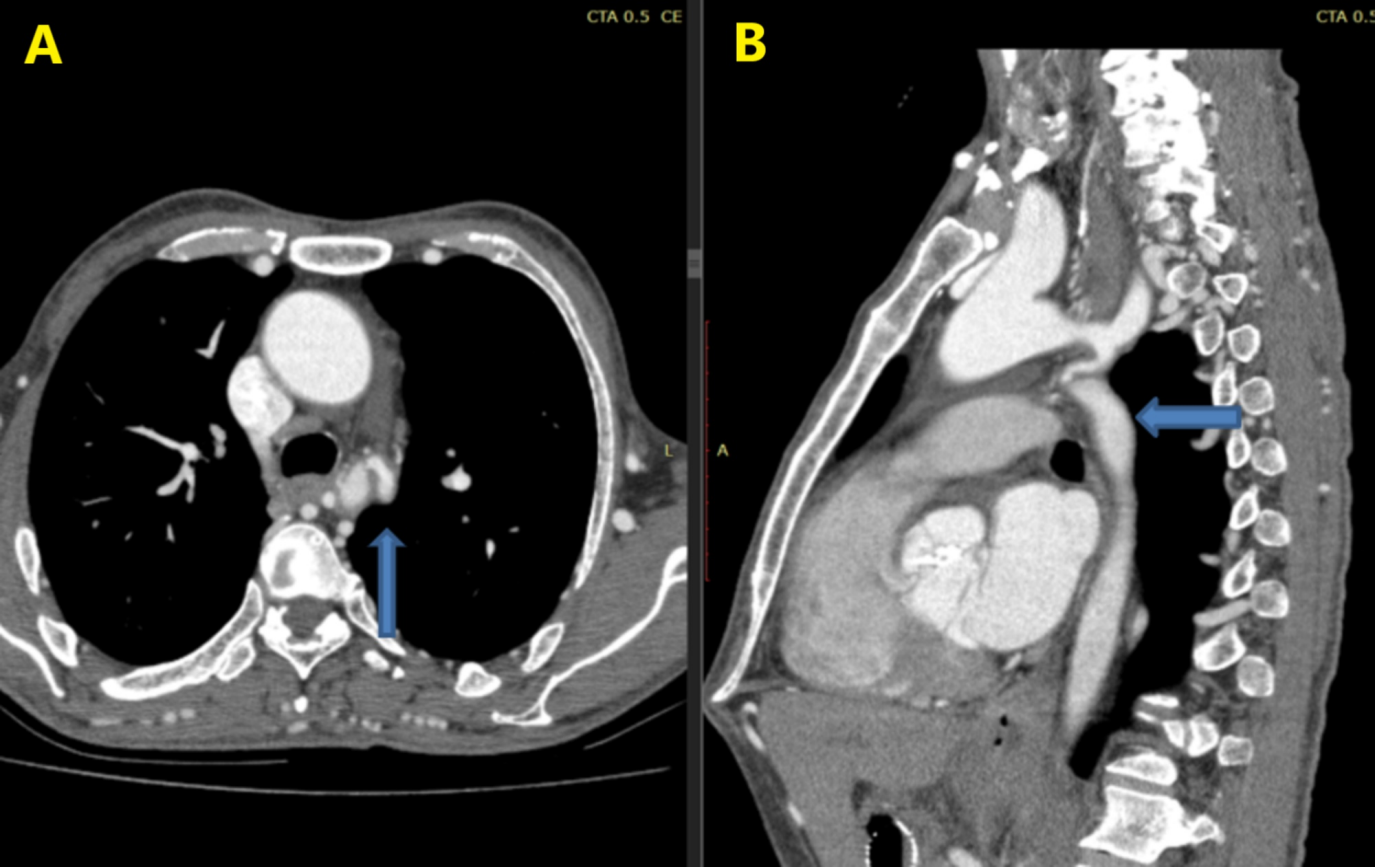
CT scan showing a narrowed segment of the aorta: (A) axial, (B) sagittal.

Thoracic CT scan revealed a narrowed segment of the aorta (Figure 1A, 1B) due to coarctation with mild dilatation of the aortic root and mild aortic stenosis. CoA may be associated with the bicuspid aortic valve, ventricular septal defect, aortic stenosis, patent ductus arteriosus, mitral valve disorders and/or intracerebral aneurysms [4]. It is typically diagnosed during childhood; however, a small proportion of patients present for the first time in adulthood. CoA is characterized by arterial hypertension in the upper body and a downstream pressure drop which is compensated by the development of extensive body wall collaterals to bypass the narrowed aorta [5].

**Figure 2:**
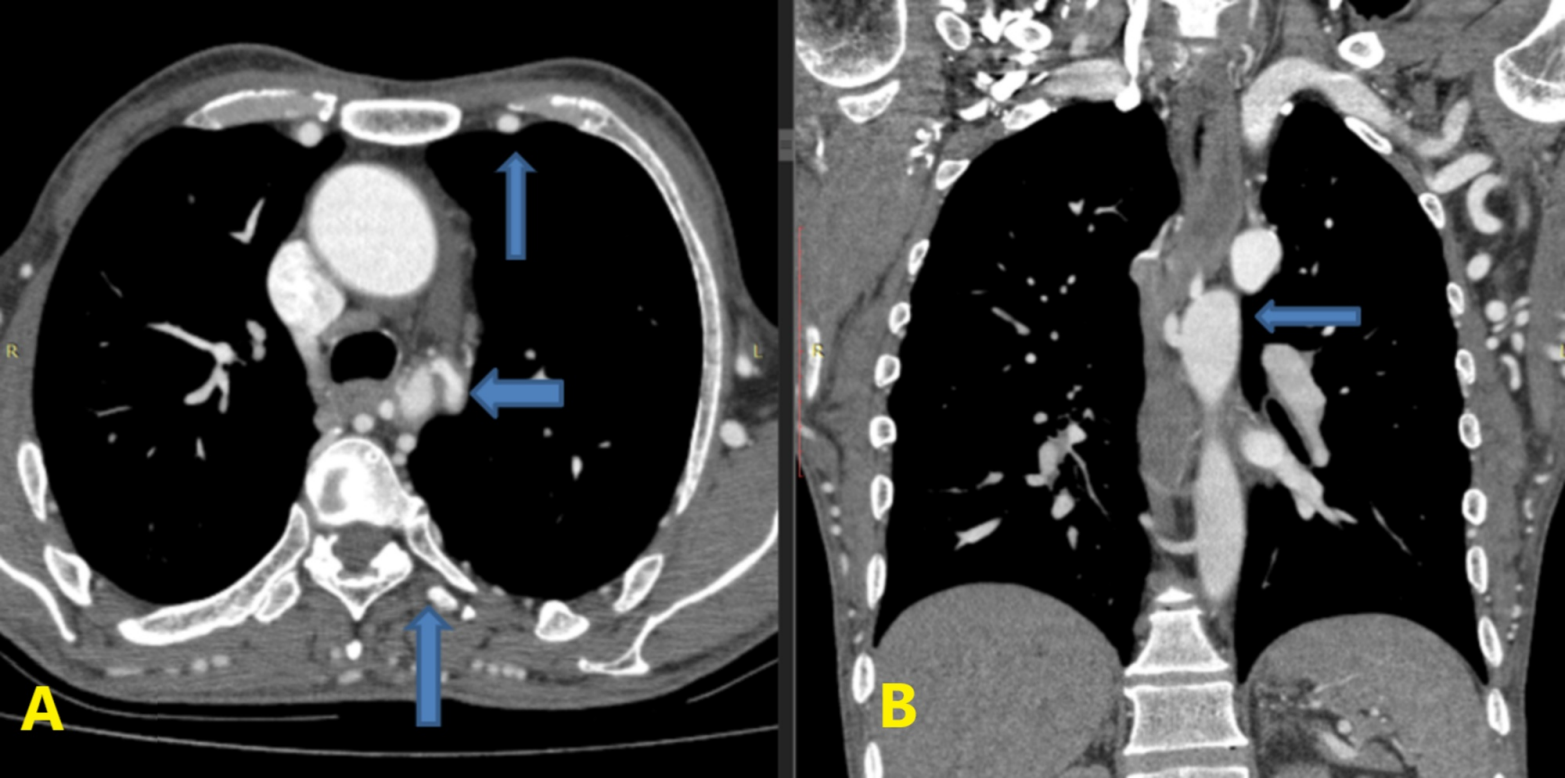
Axial CT scan showing a narrowed segment with enlarged internal thoracic and intercostal arteries (A) and coronal view demonstrating coarcted segment (B).

Survival with coarctation depends on the degree of the development of a collateral blood supply which originates from the subclavian arteries and supplies the descending aorta primarily via the intercostal, inferior epigastric and subscapular arteries [3]. However, it is poorly developed in newborns; however, it becomes increasingly robust as the patient ages. Multiple enlarged intercostals and chest wall collaterals were identified on CT scan (Figure 2A) along with the coarcted segment (Figure 2B).

**Figure 3:**
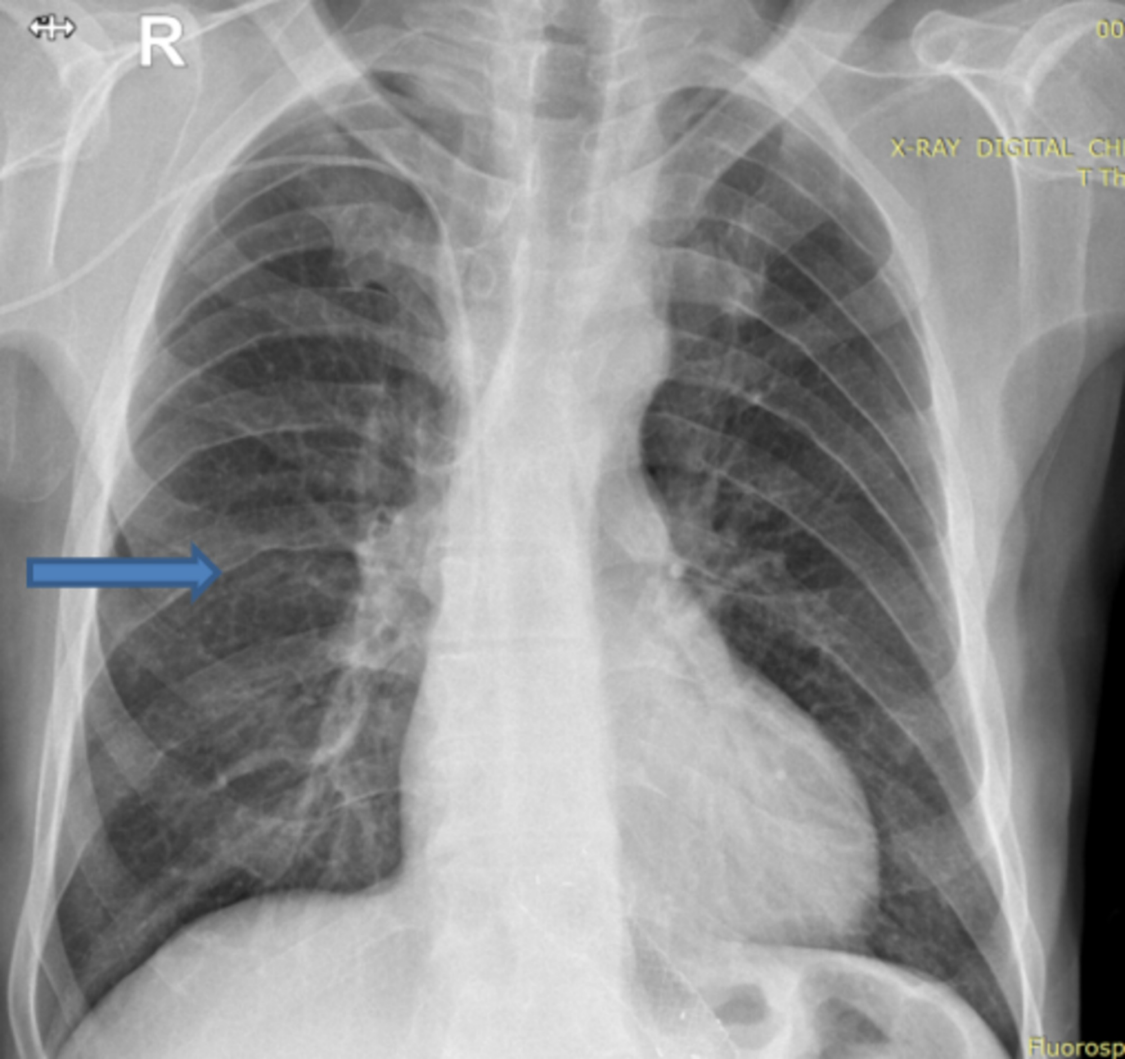
Chest X-ray revealing rib notching.

The development of rib notching is secondary to dilated intercostal collateral vessels and is commonly reported in the patients of CoA [6]. Blood supply to the ribs between the third and the ninth rib is typically affected by the coarctation; hence, notching is evident in these ribs in adults with fully formed collaterals [6,7]. The chest X-ray of the patient revealed the characteristic notching of the ribs (Figure 3).

Contrary to open surgery, laparoscopic surgery has a lower risk of cardiovascular morbidity and mortality ranging from 0.3% to 1.8% [8]. Therefore, high-risk patients frequently undergo laparoscopic surgery. However, in such high-risk patients, high intra-abdominal pressure due to CO_2_ insufflation is associated with more fluctuations in hemodynamic parameters as compared with low intra-abdominal pressure, contributing to the increase in the risk of cardiovascular complications [9]. An increase in intra-abdominal pressure results in a mechanical impairment of the venous blood return, subsequently leading to a fall in the preload and an increase in the afterload. There will be a decreased cardiac output and an increase in the systemic vascular resistance (SVR) and MAP [8]. The high MAP tends to affect the upper part of the body mainly. An increase in the SVR, secondary to the creation of pneumoperitoneum along with a decrease in stroke volume, cardiac and ejection velocity indices, can lead to further decrease in blood flow distal to the coarctation. Low-pressure pneumoperitoneum is ideal for laparoscopic surgeries as it minimizes the adverse hemodynamic effects of CO_2_ insufflation [9]. Therefore, intra-abdominal pressure should be maintained under 12 mmHg during pneumoperitoneum to minimize hemodynamic changes [10,11]. An MAP ≥ 60 mmHg is believed to be needed to maintain adequate tissue perfusion.

Anaesthetic management is aimed towards maintaining normal or slightly elevated SVR and heart rate, and maintenance of adequate intravascular volume. Invasive hemodynamic monitoring can help guide the administration of intravenous fluids. Invasive hemodynamic monitoring is required for ASA III and IV patients [11]. Surgery was performed under general anaesthesia with invasive monitoring to maintain the BP within the normal limits. Radial-femoral delay is a classical clinical sign associated with CoA [6]. It is prudent to measure the discrepancy in BP in the upper and lower limbs. We, therefore, placed two arterial lines: one in the right radial and another in right dorsalis pedis artery.

Due to extensive collateral vessels in the chest, we decided to avoid thoracoscopy or thoracotomy and performed the surgery transabdominally. We were able to achieve 3 cm proximal margin, clear of neoplasm, confirmed on final histopathology. No perioperative complication occurred, except for few episodes of hypotension secondary to the thoracic epidural which were managed with fluids and tiny phenylephrine boluses. For postoperative analgesia, epidural bupivacaine 0.125% and fentanyl 1 mcg/ml were administered to avoid pain-associated adverse effects, especially to the patient’s cardiovascular system. The epidural analgesic technique is favourable as it reduces sympathetic responses and release of catecholamines, and has a favourable effect on cardiac outcome postoperatively [12]. Moreover, 0.125% bupivacaine and fentanyl 1 mcg/ml offer good analgesia and cause minimum motor block [13].

Laparoscopic-assisted resection of the distal oesophagus and proximal stomach with oesophagogastric stapled anastomosis was successfully performed with proper anaesthetic techniques. Perioperative management goals of the patient included general anaesthesia, epidural analgesia to avoid pain-associated adverse effects and efficient control of BP distal to coarctation to limit the risk of intraoperative morbidity. The patient was discharged on the sixth postoperative day in a stable condition. He has been followed in surgical outpatient and has no postoperative complications. Since uncorrected aortic coarctation leads to significant morbidity and mortality, the patient was referred to the cardiologist for the correction of CoA.

## Conclusions

Adult patients with coarctation are frequently asymptomatic except for the incidentally noted hypertension. Therefore, it is often missed by the primary care physician during routine examination. Palpation of femoral pulses and measurement of BP during routine examination is necessary to avoid a delay in the diagnosis. In our patient, rib notching and radial-femoral delay were among the prominent features of CoA in the patient. Our case represents successful perioperative anaesthetic management of the patient with CoA undergoing surgical resection of gastric adenocarcinoma. Patients with CoA need thorough preoperative workup, and the correct choice of anaesthetic technique plays an important role in determining the outcome. Needless to mention, an efficient teamwork between cardiologist, surgeons and especially anaesthesiologists is necessary to prevent any inadvertent event intraoperatively and postoperatively. 
